# Reversal to air-driven sound production revealed by a molecular phylogeny of tongueless frogs, family Pipidae

**DOI:** 10.1186/1471-2148-11-114

**Published:** 2011-04-27

**Authors:** Iker Irisarri, Miguel Vences, Diego San Mauro, Frank Glaw, Rafael Zardoya

**Affiliations:** 1 Department of Biodiversity and Evolutionary Biology, Museo Nacional de Ciencias Naturales, CSIC, c/José Gutiérrez Abascal 2, Madrid 28006, Spain; 2 Department of Evolutionary Biology, Zoological Institute, Technical University of Braunschweig, Spielmannstrasse 8, 38106 Braunschweig, Germany; 3 Department of Zoology, The Natural History Museum, Cromwell Road, London SW7 5BD, UK; 4 Zoologische Staatssammlung München, Münchhausenstrasse 21, 81247 München, Germany

## Abstract

**Background:**

Evolutionary novelties often appear by conferring completely new functions to pre-existing structures or by innovating the mechanism through which a particular function is performed. Sound production plays a central role in the behavior of frogs, which use their calls to delimit territories and attract mates. Therefore, frogs have evolved complex vocal structures capable of producing a wide variety of advertising sounds. It is generally acknowledged that most frogs call by moving an air column from the lungs through the glottis with the remarkable exception of the family Pipidae, whose members share a highly specialized sound production mechanism independent of air movement.

**Results:**

Here, we performed behavioral observations in the poorly known African pipid genus *Pseudhymenochirus *and document that the sound production in this aquatic frog is almost certainly air-driven. However, morphological comparisons revealed an indisputable pipid nature of *Pseudhymenochirus *larynx. To place this paradoxical pattern into an evolutionary framework, we reconstructed robust molecular phylogenies of pipids based on complete mitochondrial genomes and nine nuclear protein-coding genes that coincided in placing *Pseudhymenochirus *nested among other pipids.

**Conclusions:**

We conclude that although *Pseudhymenochirus *probably has evolved a reversal to the ancestral non-pipid condition of air-driven sound production, the mechanism through which it occurs is an evolutionary innovation based on the derived larynx of pipids. This strengthens the idea that evolutionary solutions to functional problems often emerge based on previous structures, and for this reason, innovations largely depend on possibilities and constraints predefined by the particular history of each lineage.

## Background

As noticed by Darwin [1], every derived feature in an organism must have evolved from a pre-existing feature in its ancestors. Therefore, the current form and function of organism attributes are determined, to a great extent, by phyletic heritage of past events [2]. This equally applies to evolutionary key innovations, which are not designed every time anew, but use available materials, that are themselves a product of millions of years of evolution. This has been firmly established by many studies showing that (*i*) novel morphological structures may often appear by deploying ancient genetic regulatory circuits [e.g. [3]] and (*ii*) existing genes or morphological structures can be recruited to perform completely new functions [e.g. [4,5]] or to explore new approaches to carry out the same task [6]. Evolution is an integrated and unitary process [7], and effective reuse of previous molecular or morphological structures through natural selection is subjected to historical constraints [8]. Deciphering how a particular functional mechanism arises in an organism requires framing the question within an evolutionary context through a multidisciplinary approach [9] involving functional, morphological, and phylogenetic analyses that allow determining which morphological structures were involved, their evolutionary origin (i.e. homology), and the succession of steps that led to a successful end.

Sound production is a key feature in the behavior of different animals such as crickets, frogs, birds or bats [10]. Calls normally serve as advertising signals to delimit territories or attract mates, and in more sophisticated cases can become a proxy of the mood of the individual [11]. Thus, it is not surprising that such a critical function has been the subject of intensive selection through evolutionary history, and a wide variety of sound production mechanisms have evolved in different animals. In frogs, diversification of sound production mechanisms is intimately linked to and/or constrained by the evolution of vocal structures, which is necessarily connected to the evolution of the respiratory system. Despite the considerable diversity of calls and larynx morphologies among extant frogs, the majority of the species call by moving air from the lungs through the glottis [10]. In most frog species, the laryngeal apparatus, which is suspended between the posteromedial processes of the hyoid (= thyrohyals), is a cartilaginous capsule composed of two arytenoid cartilages (each bearing one vocal cord), the cricoid cartilage and associated musculature [10].

A remarkable exception to the above-described general sound production and larynx morphological patterns occurs in the family Pipidae. The extant members of this family include the South American genus *Pipa *(Surinam toads) and the four African genera *Hymenochirus, Silurana, Xenopus*, and *Pseudhymenochirus *(African clawed frogs). The family Pipidae together with its sister group, the monotypic family Rhinophrynidae (Mexican burrowing toads, genus *Rhinophrynus*), form the superfamily Pipoidea [12]. The origin of pipids dates back at least to the Mesozoic [e.g. [13-16]] with known fossils from the Cretaceous [17]. Pipids represent a nice example of highly adapted form and function that evolved from an inherited frog bauplan, which is *per se *highly specialized within amphibians (and tetrapods), and restricted to limited variation [18]. Pipids are the only fully aquatic group of frogs, and their derived morphology and biology are largely a product of adaptations to this lifestyle [19]. One of these remarkable adaptations is the pipid sound production [20], with the structure and function of their larynx being radically different from those of other frogs [20,21]. Pipids lack vocal cords, and their larynx is a greatly enlarged and (at least partially) ossified box made up by the cricoid cartilage and the tyrohyals, which do not form part of the larynx in non-pipid frogs. This box encloses the arytenoid cartilages which are modified into two bony rods [10]. The sound production mechanism was described in detail for *Xenopus borealis *[20,22], and it appears to be based on implosion of air into a vacuum formed by rapidly moving disk-like enlargements of the arytenoids. The sound is then amplified by the enlarged voice box that serves as an internal vocal sac [20,22]. Sounds thus are produced without moving an air column, and therefore without externally visible movements of the flanks or throat. Similar motionless calling was also observed in *Hymenochirus boettgeri *[23], *Pipa pipa *[21], *Pipa carvalhoi *[24], *Xenopus laevis *[25], and most other pipids [[26,27], pers. obs.]. However, *Pseudhymenochirus *was stated to produce sounds by a more conventional sound production mechanism based on moving air [20], although this behavior has so far not been documented in detail.

Despite their many derived features, in several respects pipids have been more extensively studied than any other group of frogs because *Xenopus laevis *and *Silurana tropicalis *have been used as model organisms in physiology, development, and cell and molecular biology [e.g., [28]]. Knowledge on the closest relatives of model organisms is crucial to interpret and understand the evolutionary origin of studied characters and functions, but remarkably the phylogenetic relationships of pipids have not been comprehensively assessed so far. The rather aberrant morphology of pipids was initially considered to be relatively ancestral among frogs, and many of pipid morphological characters were initially assumed to retain plesiomorphic states. However, now pipids are viewed as highly derived frogs [28] with many autapomorphies primarily related to their fully aquatic lifestyle [28]. Almost all possible alternative phylogenetic relationships among pipid genera have been recovered based on either morphological [19,29,30] or molecular [13,31,32] data sets, and the position of Pipoidea with respect to all other frog lineages remains also equally contentious [13-16,31,33]. See Additional file 1 for a detailed discussion of previously proposed hypotheses.

Here, we analyze DNA sequences of complete mitochondrial genomes and of nine nuclear genes to produce a robust phylogeny of extant pipoids. We used this phylogenetic framework to gain insights on the evolution of the sound production mechanism in pipids. In this context, we show through behavioral observations that the calling mechanism of *Pseudhymenochirus *clearly involves the movement of an air column, as it occurs in non-pipid ancestors. Given the unambiguous derived position of *Pseudhymenochirus *within pipid phylogeny, a reversal to air-driven sound production in this genus is hypothesized. In addition, we provide strong morphological evidence from comparing diverse alizarin-stained frog larynges, which show that larynx structure in *Pseudhymenochirus *has clear pipid affinities. These observations taken together, allow us to suggest that the use of air in the sound production in *Pseudhymenochirus *is an evolutionary novelty that evolved by deploying the typical larynx structures of pipids.

## Results

### New sequence data

We determined for the first time the complete nucleotide sequence of the light (L) strand of the mitochondrial (mt) genome of four pipoid frog species: *Hymenochirus boettgeri *(HM991331), *Pipa carvalhoi *(HM991332)*, Pseudhymenochirus merlini *(HM991333), and *Rhinophrynus dorsalis *(HM991334). The mt genome of *Xenopus laevis *was the first ever determined for an amphibian [34] and contained numerous minor sequencing errors probably due to technical constraints at that time. We therefore sequenced anew the full mitochondrial genome of this model species as well (HM991335). Like most metazoans [35], all five pipoid mt genomes encoded for two rRNAs, 22 tRNAs and 13 protein-coding genes, and conformed to the consensus gene order for vertebrates [35,36]. All tRNAs could be folded into the typical cloverleaf secondary structure with the known exception of *trnS-(AGY)*. The putative origin of replication of the L-strand (O_L_) was located between the *trnN *and *trnC *genes, and had the potential to fold into a stem-loop secondary structure. Three conserved sequence blocks (CSB-1, CSB-2, CSB-3) were identified in the 3' end of the mitochondrial control region in all pipoid species. Notably, our sequence of *Rhinophrynus *(a specimen from Tenexpa, Pacific coast of Mexico) differs from a previously determined sequence (GenBank accession number DQ283109; from the Caribbean coast of Texas, US) by a high uncorrected pairwise divergence of 9.9%, suggesting the existence of an unrecognized species in this monotypic genus and family.

Newly generated sequences of partial nuclear genes were deposited in GenBank under accesion numbers HM998927-HM998951, HM998953-HM998985 and HQ260710-HQ260712. Files containing the alignments of both the mitochondrial and nuclear datasets can be accessed in the Dryad Digital Repository under doi:10.5061/dryad.8962.

### Phylogenetic relationships

**Figure 1:**
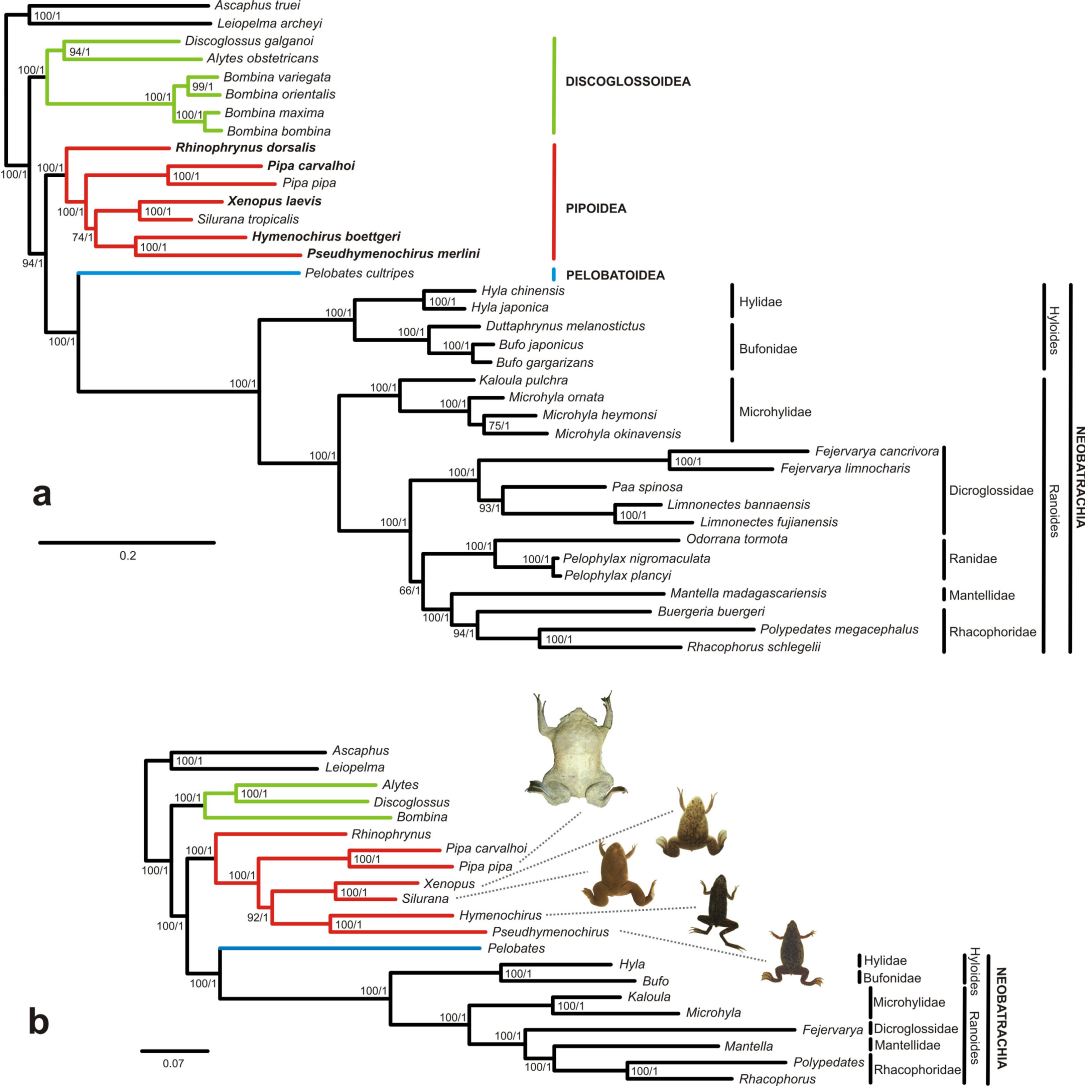
**Phylogenetic relationships among frogs**. ML trees based on concatenated DNA sequences of (a) complete mt genomes and (b) mt genomes plus nine nuclear genes. Numbers at nodes are support values from maximum likelihood bootstrap (1000 replicates; in percent) and Bayesian posterior probabilities, respectively. A congruent topology was obtained by analysis of nuclear genes only (see Additional file 1, Figure S1).

Maximum likelihood and Bayesian inference methods of phylogenetic reconstruction recovered fully congruent tree topologies for mitochondrial, nuclear, and combined datasets, respectively, with differences only in branch lengths and levels of support (Figure 1). Five major clades were recovered within Anura (Figure 1): Amphicoela (*Leiopelma *+ *Ascaphus*, which were used to root the tree), Discoglossoidea, Pipoidea, Pelobatoidea and Neobatrachia. Non-neobatrachian frogs were recovered as successively branching lineages, with Discoglossoidea branching off after Amphicoela, followed by Pipoidea and Pelobatoidea. These relationships received high support values in the analysis of mitochondrial genomes and nuclear genes, and maximum support in the combined analysis (Figure 1 and Additional file 1, Figure S1). Alternative phylogenetic placements of the Pipoidea were significantly rejected by the AU test (Table 1 and Additional file 1, Table S2).

**Table 1:** Statistical tests of alternative phylogenetic hypotheses

Phylogenetic hypotheses	-ln L	p value
Unconstrained tree	154,788	0.96
*Phylogenetic position of Pipoidea within Anura*		

Pipoidea branching before Discoglossoidea	154,827	***0.001***
Pipoidea + Pelobatoidea	154,836	***<0.001***
Pipoidea + Discoglossoidea	154,822	***0.003***
Monophyly of Archaeobatrachia	154,870	***<0.001***
*Internal relationships within Pipidae*		

(*Xenopus *+ (*Silurana *+ (*Pipa *+ (*Hymenochirus *+ *Pseudhymenochirus*))))	155,353	***<0.001***
((*Pipa *+ *Hymenochirus*) + (*Xenopus *+ *Silurana*))	154,807	0.089
*Pseudhymenochirus *basal in Pipidae	155,07	***0.021***
(*Pseudhymenochirus *+ *Hymenochirus*) basal in Pipidae	154,814	***<0.001***

Results of approximately unbiased (AU) tests based on the combined dataset of mitochondrial and nuclear DNA sequences. P-values <0.05 (bold italics) indicate that the data allow rejection of the respective alternative hypothesis.

Within the Pipoidea, all data sets and phylogenetic analyses supported *Rhinophrynus *as the sister taxon of monophyletic Pipidae, *Pipa *as sister group to all other extant pipid genera, and sister-group relationships between *Xenopus *and *Silurana*, and between *Hymenochirus *and *Pseudhymenochirus*. Alternative hypotheses could be significantly rejected except for a basal placement of the *Xenopus/Silurana *clade (Table 1). In single-gene analyses of nuclear data, *Pipa *was recovered as sister group to all other extant pipid genera by *bdnf, pomc, cxcr-4, slc8a1 *and *slc8a3*, whereas *rag1 *and *rag2 *recovered the *Xenopus/Silurana *clade or the *Hymenochirus/Pseudhymenochirus *clade in such position, respectively (Additional file 1, Table S1).

### Mechanism of sound production in *Pseudhymenochirus *and other Pipidae

**Figure 2:**
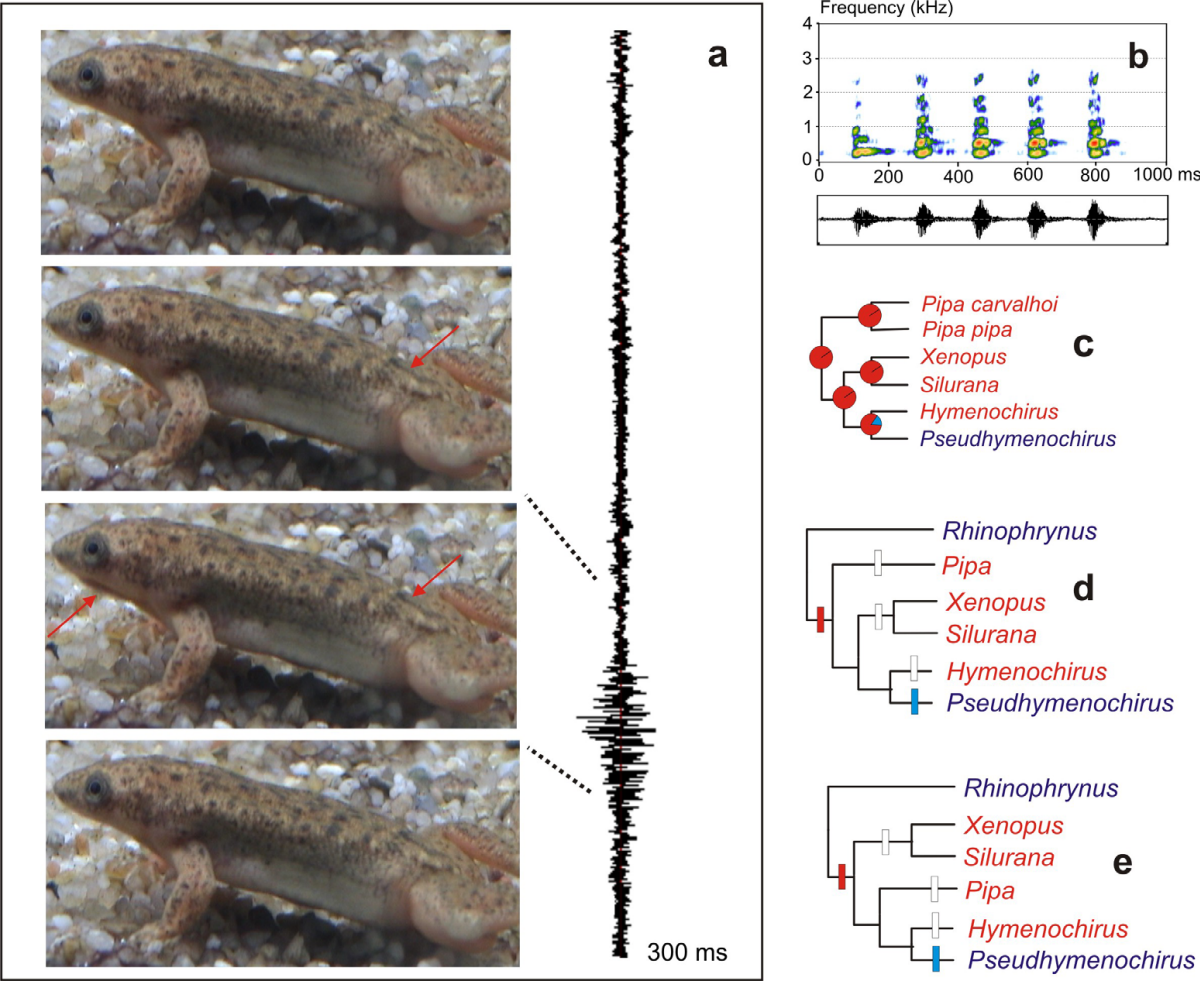
**Sound production in *Pseudhymenochirus merlini***. (a) Time series of emission of one note in a male, showing movement of throat and flanks indicative of movement of an air column (also see Aditional file 2: Movie). (b) Spectrogram and oscillogram of a male advertisement call with five notes. (c) Reconstruction under ML of ancestral character states of sound production mechanism (red without, and blue, with movement of air column) using BayesMultistate. (d) Preferred ancestral character state reconstruction of origin (red bar) and reversal (blue bar) of sound production mechanism; white bars represent the less parsimonious hypothesis of three independent origins of the implosion mechanism. (e) Same reconstruction under the alternative pipid phylogeny suggested by morphology.

In contrast to previous non-documented observations [20], we provide compelling behavioral data on *Pseudhymenochirus merlini *showing that this species, while calling, moves a column of atmospheric air from the lungs through the glottis (Additional file 2: Movie). We conclude that this movement of air almost certainly is causal for sound production in this species. Unlike all other extant pipid genera, all of which show a motionless calling, vocalizations in *Pseudhymenochirus *are clearly associated with intermittent constrictions of the posterior flanks and extension of the throat (Figure 2). The observed sequence of movements further suggests that sounds are produced during expiration, i.e., movement of the air from the lungs (Figure 2). Moreover, males produce release calls, showing also regular contractions of flanks and extension of throat.

**Figure 3:**
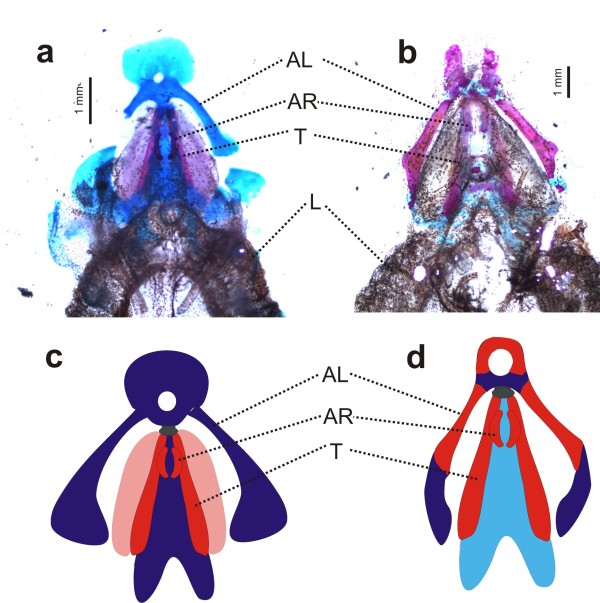
**Structure of larynx in *Pseudhymenochirus* and *Hymenochirus*.** (a) Cleared and stained preparations of the larynx of *Hymenochirus boettgeri* and (b) of *Pseudhymenochirus merlini* in dorsal view, showing a generally lower extension of cartilaginous and calcified structures surrounding the larynx in *Pseudhymenochirus*. L, lungs; AL, alary processes of hyoid plate; AR, arytenoid cartilages; T, thyrohyals (= posteromedial processes of the hyoid plate). Schematic drawings represent main larynx structures in (c) *Hymenochirus* and (d) *Pseudhymenochirus*. Colors denote calcified (red) vs non-calcified cartilaginous (blue) structures. Note the calcified alary process in Pseudhymenochirus. Modified from Ridewood [38] and Cannatella and Trueb [29].

We complemented behavioral observations with morphological comparative analyses of the larynx structures of alizarin red-alcian blue stained specimens of different pipid genera and a discoglossoid. Our results confirm previous works [29,37,38] that larynges of pipids are highly enlarged and ossified, in contrast to those of the rest of frogs. The larynx of *Xenopus*, is a highly ossified box made up by the thyrohyals, arytenoids, and cricoid cartilages, which are greatly expanded posteriorly. In *Hymenochirus *and *Pseudhymenochirus*, the larynges show conspicuous and ossified thyrohyals that enclose the smaller arytenoid rods (Figure 3). Both genera additionally share an elongate shape of lungs that reach the inguinal region and are tightly attached to the body wall (Additional file 1, Figure S4). However, there are two conspicuous differences between these two taxa: (i) *Pseudhymenochirus *has ossified alary processes of the hyoid plate which form rods very similar to the thyrohyals (= posteromedial processes of the hyoid plate) [29], and (ii) cartilage and calcified structures around the larynx are more extended and form an overall more compact laryngeal "box" structure in *Hymenochirus*, with calcified strutures lateral to the thyrohyals and extensive cartilage visible in the glottis area (Figure 3). Because all our cleared-and-stained preparations were made from adult specimens that had been sacrificed immediately previous to the clearing and staining procedure, we can exclude that preservation artefacts have caused these differences. Therefore, the larynx of *Pseudhymenochirus *seems to be more flexible, and this, somehow may allow the air to move through it.

Parsimony optimization of ancestral character states of sound production mechanism on the recovered hypothesis of pipid phylogenetic relationships, and on the only not significantly rejected alternative phylogenetic hypothesis (Figure 2) supported homoplasy of the air-driven call in *Pseudhymenochirus*. Because pipid sound production appears to be linked to the adaptation to aquatic environment [20] and character states in fossil taxa are unknown, we assume its single origin in the ancestor of Pipidae and a subsequent reversal in *Pseudhymenochirus*, i.e., two transformations. The alternative hypothesis would require assuming independent origin of the implosion mechanism in *Pipa, Xenopus *+ *Silurana*, and *Hymenochirus*, and thus three transformations. BayesMultistate reconstructed the ancestral pipid character state as using the implosion mechanism, with a ML probability >0.999 (Figure 2).

## Discussion

### Phylogenetic relationships of and within Pipoidea

Our results provided strong support for (*i*) the placement of monophyletic pipoids as the third most basal clade among extant anurans and (*ii*) the monophyly of Pipidae. These results are concordant among all data and methods of phylogenetic inference, and in agreement with previous molecular [13,14] and morphological [10,39,40] studies. Within Pipidae, our results supported the monophyly of dactylethrines (*Xenopus *and *Silurana*) as in other molecular studies [14,32] and recent morphological studies [17], and significantly rejected the previously proposed paraphyletic basal arrangement of *Xenopus *and *Silurana *[19,29]. The recovered basal position of *Pipa*, and the sister group relationship of dactylethrines and hymenochirines (*Hymenochirus *and *Pseudhymenochirus*) agrees with previously published molecular studies [13,14] but contests recent morphological analyses [17,19,29,30] that supported a Pipinae clade (including hymenochirines and *Pipa*) [17,19,29] with up to six osteological characters [after excluding fossil taxa; [17]]. The alternative hypothesis of a Pipinae clade could not be rejected by our molecular data (Table 1 and Additional file 1, Table S2). If our preferred hypothesis of pipid relationships is correct, homoplasy of the above mentioned six characters needs to be assumed. Polarization of these characters is complicated by the fact that all extant pipids are aquatic whereas their unambiguous extant sister group, *Rhinophrynus*, is a terrestrial species with specialized burrowing habits. Future morphological studies should assess additional external characters, tadpole morphology, and soft anatomy in the search for possible synapomorphies of the four African genera, such as the keratinization of the first three toes, which is more strongly expressed in the African taxa [41].

Despite the apparent contradiction to morphological data, the congruence between mitochondrial data and the various nuclear genes (Figure 1 and Additional file 1, Table S2), and the strong support for *Pipa *as sister to all other extant pipids make our conclusions considerably robust. From a biogeographic point of view our hypothesis suggests that the basal split among extant pipids might have separated an African lineage (*Hymenochirus, Pseudhymenochirus, Silurana*, and *Xenopus*) from a South American lineage (*Pipa*) and is consistent with the American distribution of Rhinophrynidae as sister group of the Pipidae [41,42]. This indicates the need of re-evaluating also the phylogeny of fossil taxa, given the apparent biogeographic anomaly that the South American *Pipa *based on morphological data are nested within a clade of purely African fossil taxa, and the African *Silurana/Xenopus *within a clade of exclusively South American fossil taxa [17].

### Evolution of sound production in Pipidae

Our behavioral observations suggest that sound production in *Pseudhymenochirus *is produced by air movement from the lungs to the throat. This is unique among pipids, which generally produce motionless clicking sounds by implosions related to the derived box-like structure of the larynx [22], and in fact more similar to the typical mechanism found in non-pipid frogs. The sound production mechanism of pipids has been thoroughly studied in *Xenopus borealis *[22] and given the resemblance of calls and motionless calling behavior in other members of the family [21,23-27], it is assumed to be the general system in pipids. In *X. borealis*, the characteristic clicking sound was proved to be produced by the simultaneous action of bipennate muscles that separate the discs of ossified arytenoid rods [22]. Yager suggested that the sound is produced by the implosion of air when the two arytenoid discs separate, given that no clicking sound was emitted when this space was filled with liquid [22]. Similarly, the implosion mechanism is not air-driven, because call spectra remained unchanged after frogs were foreced to breathe helium [22].

With regards to morphological analyses, our results are fully congruent with previous detailed anatomical descriptions in which the larynges of pipids are enlarged boxes formed by more or less ossified cartilages [20,21,37,38]. Despite the apparent diversity in larynx morphology both among pipids and among frogs, the embryological origin of involved cartilages have been traced back to the larval hyobranchial apparatus [10], leaving little doubt of their homology within amphibians [37,43]. The larynx of *Hymenochirus *is an enlarged box with ossified cartilages, more similar to that of *Xenopus *and *Silurana *than to other non-pipid anurans (laryngeal cartilages are not ossified, e.g. *Bombina *in Additional file 1, Figure S5), thus reinforcing a similar sound production system to that of *X. borealis*. In *Pipa*, although the larynx structure slightly differs from that of *Xenopus *[20,22] a similar sound production has also been suggested [21]. The larynx of *Pseudhymenochirus *is particularly similar to that of *Hymenochirus *and shows the typical ossified cartilages of other pipids [37,38]. Therefore, we could undoubtedly assert that the larynx in *Pseudhymenochirus *evolved from a typical pipid condition, but the overall structure seems to be more flexible, and this could somehow permit a movement of air that is used to vocalize, as suggested by our behavioral observations. However, whether vocal cords are present in *Pseudhymenochirus *(which are absent in pipids) or whether other strucutres are responsible for sound production requieres specific examination. Other hypotheses may also be plausible, and further detailed functional studies [as those performed by Yager; [22]] are needed in order to determine the exact mechanism through which sound is produced, as well as the precise function of involved structures. Overall, our molecular and morphological data leave neither doubt of the nested phylogenetic position of *Pseudhymenochirus *within Pipidae, nor of the clear pipid nature of its larynx. While the source used for sound production unexpectedly appear to reverse to the ancestral non-pipid condition (movement of the air column), associated anatomy evolved from a typical pipid-like larynx that likely imposed constraints to natural selection. Altogether, we suggest that the air-driven sound production in *Pseudhymenochirus *most probably represents a novel evolutionary combination and it is a remarkable example of complex anatomical modifications related to a functional shift of enormous influence in frog behavior and life history.

The selective forces for these changes are unknown, but the movements of the body flanks during the call in *Pseudhymenochirus *obviously produce water waves that might provide information about the size of the calling male to females, detected by their lateral line system. Water surface waves can play an important role in the advertisement behavior of several basal anurans [44,45]. Compared to *Hymenochirus*, sexually active *Pseudhymenochirus *males have morphologically less distinct postaxillary glands (Figure 2a), which are used in chemical communication during the breading season in *Hymenochirus *[46]. Therefore, flank movements in *Pseudhymenochirus *could serve as additional visual and mechanical signals, which might reinforce the acoustic signals to attract females and impress conspecific males.

## Conclusions

Our study exemplifies that understanding the evolutionary process underlying an innovation, here the air-driven call in *Pseudhymenochirus*, can only be achieved through an integrative comparative approach. In this particular case, behavioral observations prompted for detailed anatomical analyses, and comparative data were placed within a robust phylogenetic framework based on molecular data. Further insights on the nature of this evolutionary innovation could be gained through ontogenetic studies that disentangle how morphological constraints imposed by the rather stiff larynx box of pipids are overcome to allow the reversal to the ancestral air-driven vocalization in *Pseudhymenochirus*. The result of this study provides yet another example of how natural selection generates complex morphologies and functions by tinkering with previously available structures [47], and further reinforces the important roles of historical contingency and constraints in canalizing potential solutions to a given evolutionary problem [48].

## Methods

### Taxon sampling and DNA sequencing

We assembled a dataset of all mitochondrial genomes of frogs available from GenBank, expanded it with four (newly determined) mt genomes of additional pipoid taxa, and replaced the available sequence of *Xenopus laevis *[34] by a newly determined one from a specimen with reliable locality data. DNA was extracted using a standard phenol-chloroform protocol from voucher specimen tissue. Several overlapping fragments covering the entire mt genome were amplified by PCR using previously reported primers and cycling conditions [49]. Specific primers were also designed to amplify fragments in some species in which general primers did not work (available from authors upon request). Those fragments that contained the control region were cloned into pGEM-T vectors (Promega, Madison, WI, USA) due to observed heteroplasmy. PCR fragments and recombinant clones were cycle-sequenced with the ABI Prism BigDye Terminator Cycle Sequencing Ready Reaction Kit (V3.0) using PCR and M13 universal primers, respectively, as well as walking primers if needed. Cycle sequencing products were run on ABI Prism 3700 and 3130 × l DNA Analyzers (Applied Biosystems). The obtained mt sequences were annotated based on sequence similarity to reported frog mt genomes. The vertebrate mt genetic code was used to translate ORFs of protein-coding genes. The different tRNAs were identified based on their putative clover-leaf secondary structure, as implemented in the program DOGMA [50]. Sequences were aligned by taking secondary structure of tRNAs and amino acid translations of protein-coding genes into account. Highly variable portions of the sequences, as well as third positions of mt protein-coding genes were excluded from phylogenetic analyses (see Additional file 1 for extended methods).

A nuclear DNA dataset was generated using partial sequences of nine protein-coding genes (see Additional file 1, Table S3): *rag1, rag2, bdnf, pomc*, exon 2 of *cxcr4*, exon 2 of *slc8a1, slc8a3*, exon 1 of *rho *and *H3a*. We assembled a complete combined matrix complementing previously available sequences from GenBank with newly determined sequences, representing all major lineages of frogs for which mt genome data exist. In a few cases, chimerical sequences were constructed by merging sequences from different species of the same genera, for which strong evidence exist of being monophyletic. Primers used were as reported in the literature: *rag1 *[49]; *rag2 *[51,52]; *slc8a1 *[13]; *bdnf *and *pomc *[53]; *rho *[51]; and *H3a *[54]. In all cases, PCR cycling conditions were experimentally adjusted from those reported in the original publications.

### Phylogenetic analysis

Single-gene alignments were used to produce three different datasets, containing: (a) all mt genes (final length of 11,131 bp); (b) all nuclear genes (final length of 7,107 bp); and (c) a combination of mt and nuclear genes (final length of 18,238 bp). Additionally, we also constructed an alternative mt dataset using amino acid characters for protein-coding genes, as well as analyzed single-gene datasets for the nuclear genes to understand the congruence among these markers.

We used *Leiopelma *and *Ascaphus *as outgroup taxa because molecular and morphological data are congruent in indicating that these are the most basal extant frogs [14,55]. All datasets were subjected to maximum likelihood [ML; [56]] and Bayesian inference [BI; [57]] analyses using RAxML version 7.0.4 [58] and MrBayes version 3.1.2 [59,60], respectively. RAxML used the rapid hill-climbing algorithm [61] computing 100 distinct ML trees starting from 100 distinct randomized maximum-parsimony starting trees. BI was performed running four simultaneous Markov chains for 10 million generations, sampling every 1000 generations. An additional BI run was performed for each of the analyses, to confirm the adequate mixing of the Markov chains. Convergence was checked *a posteriori *by plots of ln*L *scores and low standard deviation of split frequencies, as well as using the convergence diagnostics implemented in the online tool AWTY [62]. The first 2.5 million generations were discarded as burn-in to prevent sampling before the Markov chains reached stationarity. Partitioned analyses were performed for ML and BI, with 16 partitions for the mitochondrial and 9 for the nuclear datasets. For each partition, the best fit-model of nucleotide substitution was chosen using the Akaike information criterion [AIC; [63]] as implemented in Modeltest version 3.7 [64], MrModeltest version 2.3 (by J. A. A. Nylander; http://www.abc.se/~nylander/), and ProtTest [65]. Support for internal branches was evaluated performing 1000 replicates of non-parametric bootstrapping [66] (ML) and by posterior probabilities (BI).

Alternative tree topologies (see results) were evaluated based on the combined mt and nuclear dataset using the non-parametric approximately unbiased (AU) test [67] as implemented in Consel version 0.1 k [68] with site-wise log-likelihoods calculated by RAxML with independent GTR + Γ + I models assigned to each of the different partitions, and one million multiscale bootstrap replicates. We used BayesMultistate [69] from the BayesTraits package (by M. Pagel and A. Meade; http://www.evolution.rdg.ac.uk/BayesTraits.html) to infer the ancestral state of the mechanism for sound production in the family Pipidae using ML.

### Cleared alizarin-stained preparations

In order to further understand the morphological basis of sound production in pipids, we performed a comparative anatomical study of the larynx structures of several pipids including *Xenopus laevis, Hymenochirus boettgeri *and *Pseudhymenochirus merlini*, as well as *Bombina bombina *as representative of non-pipid ancestors. Following standard international procedures, specimens were sacrificed using an overdosis of MS222, fixed in formalin, and differentially stained for bone and cartilage with alizarin red S and alcian blue, respectively, following a standard procedure [70].

### Behavioral observations

Observations were made of captive specimens in *ca*. 100 × 30 × 20 cm aquaria. Specimens of different pipid species were obtained from the pet trade and kept at different times between 1985-2011. All observations refer to specimens in breeding conditions, without external (hormone) stimulus. No experiments with living animals were performed. Video sequences of calling specimens of *Pseudhymenochirus *were recorded in 2010 with a Sony DCR-SR30 camera. Spectral and temporal variables of the recorded sounds were analyzed using Cooledit 96 software (Syntrillium). Sonagrams were constructed using the package seewave [71] in the R environment [72].

## Authors' contributions

II carried out molecular lab work. MV provided anatomical data. FG performed behavioral observations. II and DSM analyzed data. II, MV, DSM, FG and RZ wrote the paper. All authors read and approved the final manuscript.

## Supplementary Material

Additional file 1 **Extended background, methods and results**. It includes a detailed description of the molecular and phylogenetic reconstruction procedures, the taxon sampling strategy followed to assemble the nuclear dataset (with GenBank accession numbers and specimen vouchers), summary of previous hypotheses of phylogenetic relationships of pipids and more detailled information of results (congruent topology of combined nuclear genes, congruence among single nuclear genes and values of AU tests). It also includes an exhaustive description of the vocalizations of *Pseudhymenochirus merlini *(with sonograms) and anatomical preparations showing larynx structure of *P. merlini *and other pipids. Click here for file

Additional file 2 **Movie**. Calling male of *Pseudhymenochirus merlini*. Click here for file
