# Supplementary material for: Reversal to air-driven sound production revealed by a molecular phylogeny of tongueless frogs, family Pipidae
Source: BMC Evol Biol. 2011 Apr 27;11:114. doi: 10.1186/1471-2148-11-114 (PMC3111386; doi:10.1186/1471-2148-11-114)
Supplement: Additional file 1 — Extended background, methods and results. It includes a detailed description of the molecular and phylogenetic reconstruction procedures, the taxon sampling strategy followed to assemble the nuclear dataset (with GenBank accession numbers and specimen vouchers), summary of previous hypotheses of phylogenetic relationships of pipids and more detailled information of results (congruent topology of combined nuclear genes, congruence among single nuclear genes and values of AU tests). It also includes an exhaustive description of the vocalizations of Pseudhymenochirus merlini (with sonograms) and anatomical preparations showing larynx structure of P. merlini and other pipids. [file 1471-2148-11-114-S1.DOC]

# Additional file 1: Extended background, methods and results for the manuscript:

# Reversal to air-driven sound production revealed by a molecular phylogeny of tongueless frogs, family Pipidae

### Iker Irisarri, Miguel Vences, Diego San Mauro, Frank Glaw and Rafael Zardoya

**1. Extended molecular and phylogenetic reconstruction methods**

Total DNA was purified from muscle tissue (preserved in absolute ethanol) following proteinase k digestion, phenol-chloroform extraction, and ethanol purification [[1](#_ENREF_1)]. All PCR reactions were carried out with 5PRIME Taq DNA polymerase (5PRIME GmbH, Hamburg, Germany), except those covering the mitochondrial control regions, which used LA Taq polymerase (TaKaRa Bio Inc., Otsu, Shiga, Japan). PCR amplicons were purified by ethanol precipitation or directly purified from electrophoresis gels using the Speedtools PCR clean-up kit (Biotools B&M Labs. S.A., Madrid, Spain). DNA fragments were sequenced in an automated DNA sequencer (ABI PRISM 3700) using the BigDye Terminator v3.1 cycle-sequencing kit (Applied Biosystems, Foster City, CA, USA), and following manufacturer´s instructions. The obtained sequences averaged 700bp in length and each sequence overlapped with the next by about 50-150 bp. Differences between overlapping regions were not observed.

Individual alignments were carried out for each of the genes. In order to maximize positional homology in protein-coding genes, TranslatorX [[2](#_ENREF_2)] was used to align nucleotide sequences based on a previous alignment of their deduced amino acids [MAFFT; [3](#_ENREF_3)], and after having removed the amino acid positions of ambiguous alignment with Gblocks [[4](#_ENREF_4)]. Third codon positions of mitochondrial protein-coding genes were excluded from the final dataset due to the observed saturation, as judged by plots of pairwise uncorrected (transitions and transversions) versus corrected distances (measured as ML distances) (not shown). Ribosomal RNAs were aligned with MAFFT [[3](#_ENREF_3)] and corrected by eye for obvious misalignments. Transfer RNAs were aligned manually based on their putative secondary cloverleaf structure, and concatenated into a single dataset. Ambiguously aligned positions in both rRNA and tRNA alignments were also excluded with Gblocks v. 0.19b [[4](#_ENREF_4)].

**2. Summary of previous hypotheses of pipid relationships**

Ever since all pipid species were grouped together in a single family [[5](#_ENREF_5)], Pipidae is considered well-established, and its monophyly is supported by many synapomorphies [[6-8](#_ENREF_6)]. Traditionally, Pipidae was classified within "Archaeobatrachia" [] or "Mesobatrachia" []. Early molecular analyses based on partial mitochondrial ribosomal RNA gene sequences, supported the placement of Pipidae within a monophyletic "Archaeobatrachia" as sister group of Neobatrachia [[13-15](#_ENREF_13)]. However, morphological and recent molecular studies have strongly supported the paraphyly of non-neobatrachian frogs, even though the phylogenetic position of pipids varied among studies []. In recent literature, four main lineages of non-neobatrachian frogs are recognized: Pelobatoidea (the sister group of Neobatrachia), Pipoidea, Discoglossoidea, and the basal genera *Leiopelma* and *Ascaphus* (Amphicoela) []. Pipids are included in the clade Pipoidea [a well supported clade; [21](#_ENREF_21)], also including the monotypic family Rhinophrynidae (whose only living representative is *Rhinophrynus dorsalis*) and the fossil family Palaeobatrachidae [[23](#_ENREF_23)].

Several hypotheses have been proposed regarding the phylogenetic position of Pipoidea: (a) Pipoidea as sister of Pelobatoidea [the "Mesobatrachia" hypothesis; ]; (b) Pipoidea as sister to all other frogs []; (c) successive branching of Pipoidea + (Discoglossoidea + (Pelobatoidea + Neobatrachia)) []; (d) successive branching of Discoglossoidea + (Pipoidea + (Pelobatoidea + Neobatrachia)) []; (e) Pipoidea as sister of Discoglossoidea []; and (f) a sister group relationship of Pipoidea and Neobatrachia [].

While *Rhinophrynus* is unambiguously considered the sister taxon of Pipidae [], the phylogenetic relationships within Pipidae remain controversial, and many alternative hypotheses have been proposed for the relationships of the five recognized genera: (a) (*Xenopus* + (*Silurana* + (*Pipa* + (*Hymenochirus* + *Pseudhymenochirus*)))) []; (b) ((*Pipa* + *Hymenochirus*) + (*Xenopus* + *Silurana*)) []; (c) (*Hymenochirus* + (*Pipa* + (*Xenopus* + *Silurana*))) [[21](#_ENREF_21)]; (d) (*Pipa* + (*Hymenochirus* + (*Xenopus* + *Silurana*))) [[18](#_ENREF_18)]. The latter hypothesis is also consistent with other studies with a smaller taxon sampling [].

The monotypic genus *Pseudhymenochirus* has been poorly studied in the past due to its rarity in collections [[33](#_ENREF_33)]. Initially, it was grouped with *Hymenochirus* [[39](#_ENREF_39)], but later regarded as "intermediate" between *Hymenochirus* and *Xenopus* [] or considered a "primitive" *Hymenochirus* [[42](#_ENREF_42)] and finally accepted as sister group of *Hymenochirus* [[33](#_ENREF_33)]. Geographically, *Pseudhymenochirus* *merlini* is separated by 2000 km from the westernmost *Hymenochirus* species in Nigeria [[43](#_ENREF_43)].

**3. Phylogenetic relationships of frogs based on nuclear genes and AU tests**

A concatenated data set including nuclear *rag1*, *rag2*, *bdnf*, *pomc*, exon 2 of *cxcr4*, exon 2 of *slc8a1. slc8a3*, exon 1 of *rho* and *H3a* gene sequences was used to reconstruct frog phylogenetic relationships. The reconstructed tree is shown in Additional file 1, Figure S1.


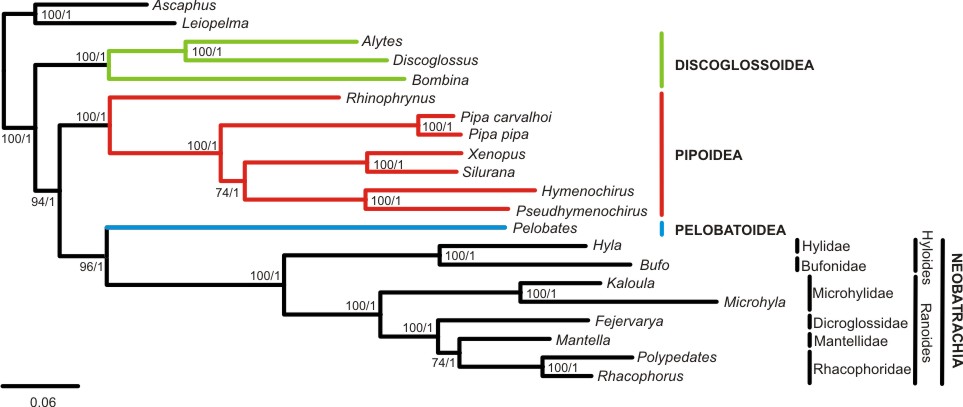


**Figure S1.** Phylogenetic relationships (ML phylogram) among frogs suggested by the analysis of concatenated DNA sequences of nine nuclear genes. Numbers at nodes are support values from maximum likelihood bootstrap (1000 replicates; in percent) and Bayesian posterior probabilities.

**Table S1**. Summary of support for phylogenetic relationships among pipoids from single-gene ML bootstrap analyses of seven nuclear genes (data for two additional genes, rhodopsin and histone 3, is not shown because the analyzed fragments were very short). Strong support refers to proportions of non-parametric bootstrapping > 70%. Monophyly of *Pipa* refers to the monophyly of *P. carvalhoi* and *P. pipa* when the sequences of both species were available for the individual gene analyses, otherwise a hyphen is shown.

|  | ***rag-1*** | ***rag-2*** | ***bdnf*** |
| --- | --- | --- | --- |
| **Monophyly of Pipoidea** | strong support | strong support | not recovered |
| **Monophyly of Pipidae** | strong support | strong support | strong support |
| **Internal relationships within Pipidae** | Dactylethrinae is basal;  weak support | Hymenochirini is basal; weak support | *Pipa* is basal;  weak support |
| **Monophyly of *Pipa*** | strong support | - | - |
| **Dactylethrinae: *Xenopus* + *Silurana*** | strong support | strong support | strong support |
| **Hymenochirini:**  ***Hymenochirus* + *Pseudhymenochirus*** | strong support | strong support | strong support |

|  | ***slc8a1,* exon 2** | ***pomc*** | ***cxcr-4,* exon 2** | ***slc8a3*** |
| --- | --- | --- | --- | --- |
| **Monophyly of Pipoidea** | strong support | not recovered | strong support | strong support |
| **Monophyly of Pipidae** | strong support | strong support | strong support | strong support |
| **Internal relationships within Pipidae** | *Pipa* is basal;  weak support | *Pipa* is basal;  weak support | *Pipa* is basal; strong support | *Pipa* is basal;  weak support |
| **Monophyly of *Pipa*** | strong support | - | - | - |
| **Dactylethrinae: *Xenopus* + *Silurana*** | strong support | strong suport | strong support | strong support |
| **Hymenochirini:**  ***Hymenochirus* + *Pseudhymenochirus*** | strong support | strong suport | strong support | strong support |

Statistical support from the combined mitochondrial + nuclear data set of alternative hypotheses of frog relationships from the literature was evaluated with the AU test:

**Table S2.** Results of the approximately unbiased (AU) test using the combined matrix with all the 37 mitochondrial and nine nuclear genes. References of the alternative hypotheses are given below each tested topology.

| **Alternative hypotheses** | **-ln L** | **p value** |
| --- | --- | --- |
| *Phylogenetic position of Pipoidea within Anura* | | |
| Unconstrained tree | 154,788.1339 | 0.96 |
| Pipoidea branching before Discoglossoidea  [] | 154,826.5114 | 0.001 |
| Pipoidea + Pelobatoidea  [] | 154,836.3637 | 2·10^5^ |
| Pipoidea + Discoglossoidea  [] | 154,821.8280 | 0.003 |
| Monophyly of Archaeobatrachia  (Pelobatoidea+(Pipoidea+((*Leiopelma*+*Ascaphus*)+Discoglossoidea))))+Neobatrachia)) [[14](#_ENREF_14)] | 154,869.7927 | 4·10^6^ |
| *Internal relationships within Pipidae* | | |
| (*Xenopus* + (*Silurana* + (*Pipa* + (*Hymenochirus* + *Pseudhymenochirus*))))  [[33](#_ENREF_33)] | 155,352.7071 | 1·10^9^ |
| ((*Pipa* + *Hymenochirus*) + (*Xenopus* + *Silurana*))  [] | 154,807.3520 | 0.089 |
| *Pseudhymenochirus* basal in Pipidae | 155,079.4269 | 0.021 |
| (*Pseudhymenochirus* + *Hymenochirus*) basal in Pipidae | 154,813.8851 | 1·10^6^ |

**4. Description of the vocalizations of *Pseudhymenochirus merlini***

We could observe two different types of vocalizations in *P. merlini:* male advertisement calls and release calls. Male advertisement calls were emitted underwater; whereas release calls were emitted when the observer gently clasped a male in the inguinal region. No female calls were heard, and no female release calls could be evoked when clasping unreceptive females, despite several attempts in different specimens.

Advertisement calls were emitted by submerged males sitting on the ground of the aquarium, in a posture with the head slightly turned upwards. During sound emissions, weak but very distinctly recognizable contractions of the flanks, especially in the inguinal region, occurred, alternating with a slight inflation and deflation of the throat. One sequence started with the contraction of the flanks, and subsequently the throat became inflated. During this sequence, one note was emitted, clearly indicating an expiratory sound production mechanism in which sound production relies on the air stream running from the lungs to the throat (see Additional file 2: Movie). The advertisement call is a rapid series of usually four, sometimes five, short non-melodious notes. The below description is based on recordings of a single male (from Guinea-Bissau, western Africa) without hormonal stimulation, but other males were observed to emit similar calls. Call duration in four-note calls is 604-682 ms (mean 642 ± 22 ms; N=20), interval between calls 1930-3225 ms (2392 ± 363 ms; N=20). Note duration is 23-35 ms (29 ± 4 ms; N=20, measured on 7 different calls), duration of intervals between notes is 127-158 ms (138 ± 12 ms; N=20). No clear pulses can be recognized within each note. Frequency is 50-2200 Hz, dominant frequency about 690 Hz.


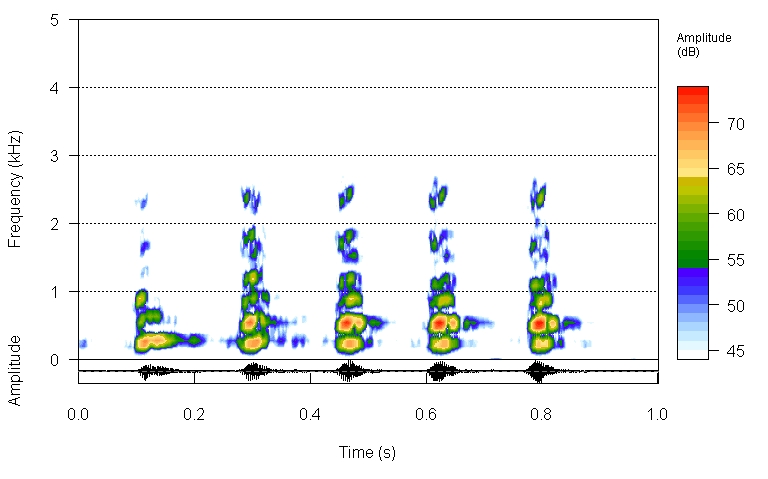


**Figure S2.** Sonagram and oscillogram of one advertisement call (with five notes) of *Pseudhymenochirus merlini*.

Release calls were regularly emitted by a male when clasped. They are short series of rather irregular pulsed notes of variable duration. In one such release call, note duration was ca. 120-190 ms (exact limits between notes were difficult to define). Frequency was 1500-5000 Hz, with some bands also recognizable up to > 10000 Hz. Dominant frequency was 2550 Hz. Notes contained about 10-20 distinct pulses which often were arranged in two pulse groups. During the call, flank contractions were observed, suggesting that the sound is indeed produced by an airstream mechanism. Pulse rate was about 140 per second. The call strongly reminded the advertisement calls of painted frogs of the genus *Discoglossus* which have two distinct pulse groups corresponding to an inspiratory and expiratory airstream [].


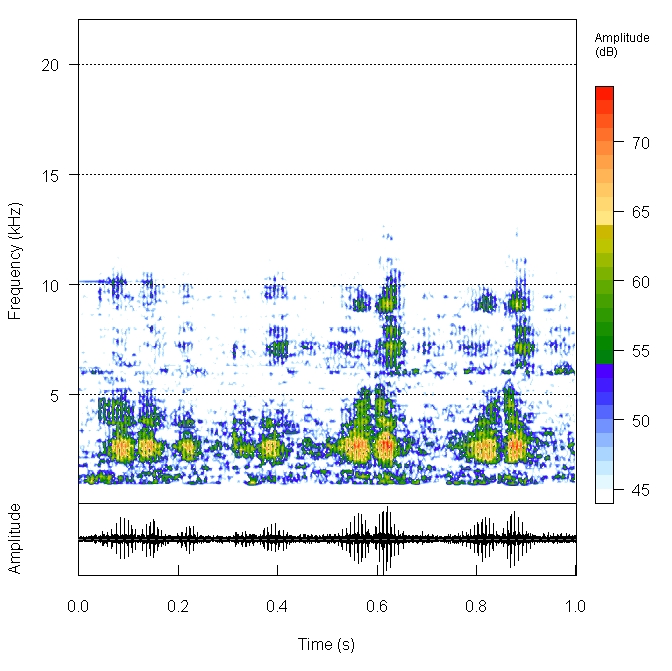


**Figure S3.** Sonagram and oscillogram of a release call (with five notes) of *Pseudhymenochirus merlini*.

**5. Larynx structure in *Pseudhymenochirus merlini* and other pipids**

A detailed comparative anatomical and functional analysis of the larynx of *Pseudhymenochirus* is beyond the scope of the present paper. However, on the basis of fresh dissections and anatomical preparations of fixed specimens with differential bone and cartilage staining of males of *Pseudhymenochirus*, *Hymenochirus, Xenopus,* and various non-pipid frogs (including *Bombina,* as well as neobatrachians), we illustrate several key points to further understand the call mechanism observed in *Pseudhymenochirus*.

First of all, we verified that larynx in pipids is a prominent box-like structure surrounded by hard cartilage, which is (at least partially) ossified [[46-48](#_ENREF_46)], in contrast all other non-pipid frogs (e.g. *Bombina*, Additional file 1, Figure S5). We also confirmed previous anatomical descriptions of *Xenopus* [] and *Hymenochirus* [[48](#_ENREF_48)]. Furthermore, upon fresh anatomical dissections*,* we could stimulate the production of single clicks in the isolated larynx of *X. laevis* by gently touching and pressing the tendon muscles simultaneously on both sides of the larynx capsule, similar to what has been described for *X. borealis* [[49](#_ENREF_49)].


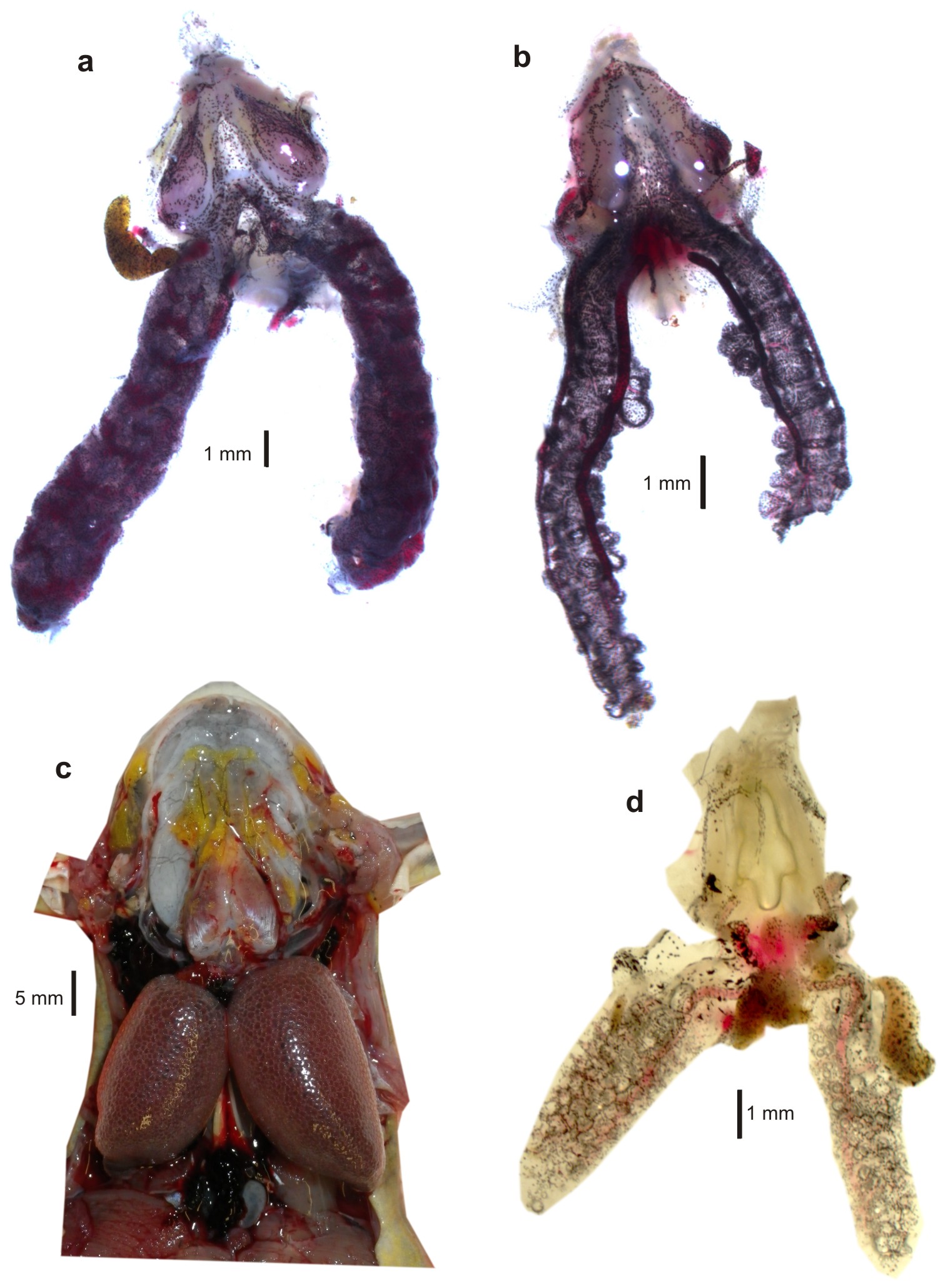
Our morphological evidence clearly shows the pipid nature of the larynx of *Pseudhymenochirus.* At first glance (Additional file 1, Figure S4 and S5), it forms an enlarged box-like structure, very similar to that of *Hymenochirus*, and they share elongated and tubular lungs that reach the inguinal region and are tightly connected to the body wall (Additional file 1, Figure S4). The overall pipid-like larynx in *Pseudhymenochirus* is clearly illustrated by the presence of the typical modified and ossified arytenoid cartilages and thyrohyals of pipids []. However, the larynx of *Pseudhymenochirus* appear much less robust than that of its sister genus *Hymenochirus* (Additional file 1, Figure S5), and thus we suggest that this fact would make the overall larynx more flexible and somehow permit a movement of air through it to produce vocalizations. However, whether vocal cords, which are absent in other pipids [[46-48](#_ENREF_46)], are present in *Pseudhymenochirus,* or whether different structures are responsible for sound production during movement of the airstream requires further detailed examination

**Figure S4.** Fresh preparations of larynx and lungs in (a) *Pseudhymenochirus merlini,* (b) *Hymenochirus boettgeri*, and (c) adult and (d) juvenile *Xenopus laevis.* Note similarity in the elongate form of the lungs and superficially box-like larynx structure between *Hymenochirus* and *Pseudhymnochirus*.


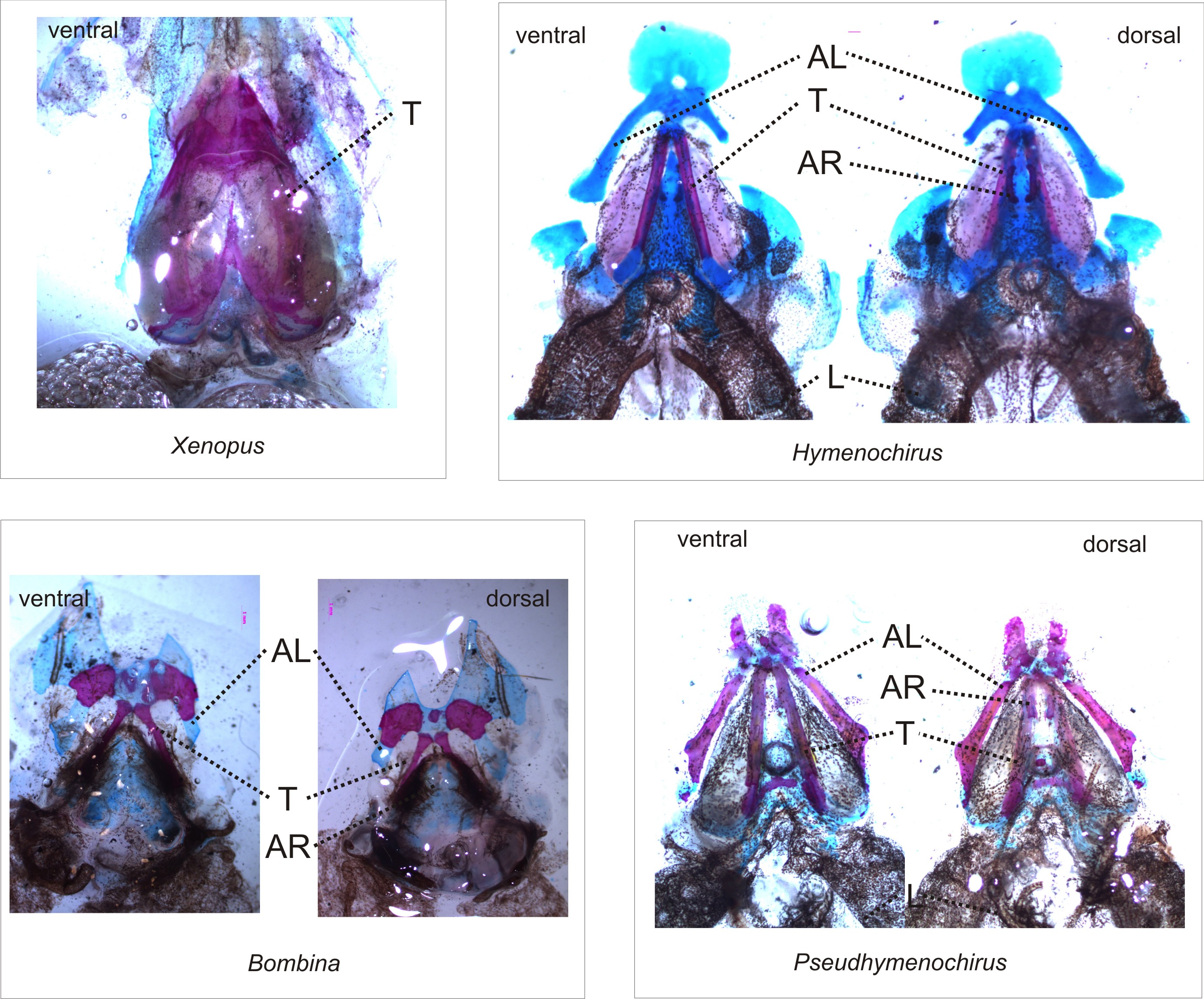


**Figure S5.** Alizarin red-alcian blue stained and cleared preparations of the larynges of the pipid frogs *Pseudhymenochirus merlini, Hymenochirus boettgeri*, and *Xenopus laevis,* and the discoglossoid frog *Bombina orientalis*. Abbreviations: L, lungs; AL, alary processes of the hyoid plate; AR, arytenoid cartilages; T, thyrohyals (= posteromedial processes of hyoid). Note that in the three pipids, the whole box-like structure with numerous calcified elements (red stain) is the larynx (not marked), whereas the larynx of *Bombina* only consists of cartilaginous elements (blue stain) and soft tissue. In *Bombina* the thyrohyals are not directly connected to the larynx while in the pipids it is an integral part of the box-like larynx structure. In *Xenopus* the larynx is a fully calcified box whereas in *Hymenochirus* it is at least laterally calcified, probably by extensions of the thyrohyals, and furthermore closed by cartilage. In contrast, no calcified extensions of thyrohyals are visible in *Pseudhymenochirus,* and cartilage is less prominent, leading us to hypothesize that the general larynx structure is probably more flexible.

**References**

1. Sambrook J, Fritsch EF, Maniatis T: **Molecular cloning: A laboratory manual**. Cold Spring Harbor, New York: Cold Spring Harbor Laboratory Press; 1989.

2. Abascal F, Zardoya R, Telford MJ: **TranslatorX: Multiple alignment of nucleotide sequences guided by amino acid translations**. *Nucl Acids Res* 2010, **38**(suppl_2):W7-W13.

3. Katoh K, Misawa K, Kuma K-i, Miyata T: **MAFFT: A novel method for rapid multiple sequence alignment based on fast Fourier transform**. *Nucl Acids Res* 2002, **30**(14):3059-3066.

4. Castresana J: **Selection of conserved blocks from multiple alignments for their use in phylogenetic analysis**. *Mol Biol Evol* 2000, **17**(4):540-552.

5. Noble GK: **The phylogeny of the Salientia. I. The osteology and the thigh musculature: Their bearing on classification and phylogeny**. *Bull Am Mus Nat Hist* 1922, **46**:1-87.

6. Ford L, Cannatella DC: **The major clades of frogs**. *Herpetol Monogr* 1993, **7**:93-117.

7. Haas A: **Phylogeny of frogs as inferred from primarily larval characters (Amphibia: Anura)**. *Cladistics* 2003, **19**:23-89.

8. Cannatella DC, Trueb L: **Evolution of pipoid frogs: Intergeneric relationships of the aquatic frog family Pipidae (Anura)**. *Zool J Linn Soc* 1988, **94**:1-38.

9. Reig OA: **Propositions for a new macrosystematics of anurans (preliminary note)**. *Physis* 1958, **21**:109-118.

10. Duellman WE: **On the classification of frogs**. *Occ Pap Mus Nat His Univ Kansas* 1975, **42**:1-14.

11. Laurent RF: **A sketch of anuran phylogeny**. *Bull Soc Zool Fr* 1979, **104**:397-422.

12. Dubois A: **Miscellanea nomenclatorica batrachologica (VII)**. *Alytes* 1985, **4**(2):61-78.

13. Hedges SB, Maxson LR: **A molecular perspective on lissamphibian phylogeny**. *Herpetol Monogr* 1993, **7**:27-42.

14. Hay JM, Ruvinsky I, Hedges SB, Maxson LR: **Phylogenetic relationships of amphibian families inferred from DNA sequences of mitochondrial 12S and 16S ribosomal RNA genes**. *Mol Biol Evol* 1995, **12**(5):928-937.

15. Dutta SK, Vasudevan K, Chaitra MS, Shanker K, Aggrwal RK: **Jurassic frogs nd the evoution of amphibian endemism in the Western Ghats**. *Curr Sci* 2004, **86**(1):211-216.

16. Gissi C, San Mauro D, Pesole G, Zardoya R: **Mitochondrial phylogeny of Anura (Amphibia): A case study of congruent phylogenetic reconstruction using amino acid and nucleotide characters**. *Gene* 2006, **366**:228-237.

17. Hoegg S, Vences M, Brinkmann H, Meyer A: **Phylogeny and comparative substitution rates of frogs inferred from sequences of three nuclear genes**. *Mol Biol Evol* 2004, **21**(7):1188-1200.

18. Roelants K, Bossuyt F: **Archaeobatrachian paraphyly and Pangaean diversification of crown-group frogs**. *Syst Biol* 2005, **54**(1):111-126.

19. Roelants K, Gower DJ, Wilkinson M, Loader S, Biju SD, Guillaume K, Moriau L, Bossuyt F: **Global patterns of diversification in the history of modern amphibians**. *Proc Natl Acad Sci USA* 2007, **104**(3):887-892.

20. San Mauro D, Vences M, Alcobendas M, Zardoya R, Meyer A: **Initial diversification of living amphibians predated the breakup of Pangaea**. *Am Nat* 2005, **165**(5):590-599.

21. Frost DR, Grant T, Faivovich J, Bain RH, Haas A, Haddad CFB, de Sá RO, Channing A, Wilkinson M, Donnellan SC, Raxworthy CJ, Campbell, JA, Blotto BL, Moler P, Drewes RC, Nussbaum RA, Lynch JD, Green DM, Wheeler WC: **The amphibian tree of life**. *Bull Am Mus Nat Hist* 2006, **297**:1-370.

22. Cannatella DC: **An Integrative phylogeny of Amphibia**. In: *Hearing and Sound Communication in Amphibians.* Edited by Peter M. Narins ASF, Richard R. Fay and Arthur N. Popper, vol. 28. New York: Springer; 2006: 12-43.

23. Spinar ZV: **Tertiary frogs from central Europe**. The Hague; 1972.

24. García-París M, Buchholz DR, Parra-Olea G: **Phylogenetic relationships of Pelobatoidea re-examined using mtDNA**. *Mol Phylogenet Evol* 2003, **28**:12-23.

25. Hillis DM, Ammerman LK, Dixon MT, de Sá RO: **Ribosomal DNA and the phylogeny of frogs**. *Herpetol Monogr* 1993, **7**:1118-1200.

26. Púgener LA, Maglia AM, Trueb L: **Revisiting the contribution of larval characters to an analysis of phylogenetic relationships of basal anurans**. *Zool J Linn Soc* 2003, **139**:129-155.

27. Starrett PH: **Evolutionary patterns in larval morphology**. In: *Evolutionary biology of the anurans: Contemporary research on major problems.* Edited by Vial JL. Columbia: University of Misouri Press; 1973: 251-271.

28. Lynch JD: **The transition from archaic to advanced frogs**. In: *Evolutionary biology of the anurans: Contemporary research on major problems.* Edited by Vial JL. Columbia: University of Missouri Press; 1973: 133-182.

29. Duellman WE, Trueb L: **Biology of amphibians**. New York: MacGraw-Hill; 1986.

30. Haas A: **The larval hyobranchial apparatus of discoglossoid frogs: Its structure and bearing on the systematics of the Anura (Amphibia: Anura)**. *J Zool Syst Evol Res* 1997, **35**(4):179-197.

31. Irisarri I, San Mauro D, Green DM, Zardoya R: **The complete mitochondrial genome of the relict frog *Leiopelma archeyi*: Insights into the root of the frog Tree of Life**. *Mitochondrial DNA* 2010, **21**(5):173-182.

32. Kjer KM: **Use of rRNA secondary structure in phylogenetic studies to identify homologous positions: An example of alignment and data presentation from the frogs**. *Mol Phylogenet Evol* 1995, **4**(3):314-330.

33. Cannatella DC, Trueb L: **Evolution of pipoid frogs: Morphology and phylogenetic relationships of *Pseudhymenochirus***. *J Herpetol* 1988, **22**(4):439-456.

34. Maglia AM, Púgener LA, Trueb L: **Comparative development of anurans: Using phylogeny to understand ontogeny**. *Am Zool* 2001, **41**(3):538-551.

35. Evans BJ, Kelley DB, Melnick DJ, Cannatella DC: **Evolution of *rag-1* in polyploid clawed frogs**. *Mol Biol Evol* 2005, **22**(5):1193-1207.

36. Evans BJ, Kelley DB, Tinsley RC, Melnick DJ, Cannatella DC: **A mitochondrial DNA phylogeny of African clawed frogs: Phylogeography and implications for polyploid evolution**. *Mol Phylogenet Evol* 2004, **33**(1):197-213.

37. Báez AM, Trueb L: **Redescription of the paleogene *Shelania pascuali* from Patagonia and its bearing on the relationships of fossil and recent pipoid frogs**. *Sci Pap Nat Hist Mus Univ Kansas* 1997, **4**:1-41.

38. de Sá RO, Hillis DM: **Phylogenetic relationships of the pipid frogs *Xenopus* and *Silurana*: An integration of ribosomal DNA and morphology**. *Mol Biol Evol* 1990, **7**(4):365-376.

39. Chabanaud P: **Contribution to the study of the herpetological fauna of West Africa**. *Bull Com Études Hist Scient Afr Occid Franç* 1921:445-472.

40. Dunn ER: **American frogs of the family Pipidae**. *Am Mus Novit* 1948, **1384**:1-13.

41. Noble GK: **The biology of Amphibia**. New York: Dover Publishing; 1931.

42. Sokol OM: **The free swimming *Pipa* larvae, with a review of pipid larvae and pipid phylogeny (Anura: Pipidae)**. *J Morphol* 1977, **154**(3):357-425.

43. Menzies JI: **An ecological note on the frog *Pseudhymenochirus merlini* Chabanaud in Sierra Leone**. *J W African Sci Ass* 1967, **12**:23-28.

44. Weber R: **Comparative studies on the bioacoustics of *Discoglossus pictus* Otth 1837 and *D. sardus* Tschudi 1837 (Discoglossidae, Anura)**. *Zool Jb Physiol* 1974, **18**:40-84.

45. Glaw F, Vences M: **Bioacoustic differentiation in painted frogs (*Discoglossus*)**. *Amphibia-Reptilia* 1991, **12**:385-394.

46. Ridewood WG: **On the structure and development of the hyobranchial skeleton and larynx in *Xenopus* and *Pipa*, with remarks on the affinities of the Aglossa**. *Zool J Linn Soc* 1898, **26**:53-128.

47. Yager DD: **A unique sound production mechanism in the pipid anuran *Xenopus* *borealis***. *Zool J Linn Soc* 1992, **104**(4):351-375.

48. Ridewood WG: **On the hyobranchial skeleton and larynx of the new aglossal toad, *Hymenochirus boettgeri***. *Zool J Linn Soc* 1899, **27**:454-460.

49. Yager DD: **Sound production and acoustic communication in *Xenopus borealis*.** In: *The Biology of* Xenopus*.* Edited by Tinsley RC, Kobel HR. Oxford: Clarendom Press; 1996: 121-141.

50. Rabb GB: **On the unique sound production of the Surinam toad, *Pipa pipa***. *Copeia* 1960, **4**:368-369.

**Table S3.** Taxon sampling strategy and GenBank accession numbers of sequences used to construct the nuclear dataset of nine nuclear loci. Newly generated data for this study has shaded cells . The number below each gene name represents the length of the alignment. If different species of the same genus were merged to form composite taxa, species are specified for each of the gene sequences.

Voucher specimens and localities of specimens used for sequencing of nuclear genes: *Leiopelma archeyi* (RM2215; Whareorino forest, west of Te Kuiti, New Zealand), *Ascaphus* *truei* (MNCN/ADN 28468; Flathead Creek, Glacier National Park, Montana, USA), *Bombina* *orientalis* (MNCN/ADN 4314; unknown locality, pet trade), *Discoglossus galganoi* (MNCN/AND 4315; Reliegos, Spain), *Alytes* *dickhillenii* (MNCN/ADN 28461; Spain), *Rhinophrynus* *dorsalis* (MNCN/ADN 28469; Tenexpa, Guerrero, Mexico), *Pipa* *carvalhoi* (MNCN/ADN 28466; unknown locality), *Xenopus* *laevis* (MNCN/ADN 28464; Jonkershoek, South Africa), *Hymenochirus* *boettgeri* (MNCN/ADN 28465; unknown locality, pet trade), *Pseudhymenochirus* *merlini* (MNCN/ADN 28467; bred in captivity, parents from ca. 130 km east of the capital Bissau, Guinea Bissau), *Pelobates* *fuscus* *fuscus* (ACZC0053; Turin, Italy), *Duttaphrynus melanostictus* (ZCMV11016; unknown locality, pet trade), *Hyla* *chinensis* (ZCMV11019; unknown locality, pet trade), *Microhyla* sp. (MNCN/ADN 28462; unknown locality), *Kaloula* *pulchra* (ZCMV11017; unknown locality, pet trade), *Fejervarya* *limnocharis* (MNCN/ADN 28470; Sri Lanka), *Mantella* *madagascariensis* (IABH6960; unknown locality), *Polypedates* *cruciger* (MNCN/ADN 28463; unknown locality, pet trade) and *Rhacophorus* *dennnysi* (ZCMV11011; unknown locality, pet trade).

(ACZC; *Zoological Collection of Angelica Crottini, Italy*; MNCN/ADN, DNA and tissue collection, *Museo Nacional de Ciencias Naturales, Spain*; IABH, *Institute for Amphibian Biology of Hiroshima, Japan*; RM, *Redpath Museum, Canada*; ZCMV, *Zoological Collection of Miguel Vences, Germany*)

| **SPECIES** | ***rag1*** | ***rag2*** | ***bdnf*** | ***slc8a1* exon 2** | ***pomc*** | ***rho* exon1** | ***H3a*** | ***cxcr-4* exon 2** | ***slc8a3*** |
| --- | --- | --- | --- | --- | --- | --- | --- | --- | --- |
|  | **1512bp** | **807bp** | **696bp** | **1272bp** | **507bp** | **309bp** | **321bp** | **675bp** | **1125bp** |
| *Leiopelma archeyi* | HM998973 | HM998978 | HM998927 | HM998951 | HM998959 | DQ283895 | HM998942 | AY523700 | EF107408 |
| *Ascaphus truei* | AY323754 | HM998977 | EU275896 | AY523731 | EU275850 | AY323730 | DQ284162 | AY523695 | AY948893 |
| *Bombina orientalis* | AY583335 | AY323783 | HM998928 | AY523715 | AY692246 | HM998984 | HM998943 | 37724434 | AY948867 |
| *Discoglossus* | *D. galganoi* | *D. sardus* | *D. galganoi* | *D. pictus* | *D. galganoi* | *D. galganoi* | *D. galganoi* | *D. pictus* | *D. pictus* |
|  | AY583338 | AY323785 | HM998929 | AY523708 | HM998960 | DQ283915 | HM998944 | AY364172 | AY948858 |
| *Alytes* | *A. obstetricans* | *A. muletensis* | *A. dickhilleni* | *A. obstetricans* | *A. dickhilleni* | *A. obstetricans* | *A. dickhilleni* | *A. obstetricans* | *A. obstetricans* |
|  | AY583334 | AY323781 | EF407511 | Y523703 | HM998961 | DQ283825 | HM998945 | AY364170 | EF107345 |
| *Rhinophrynus dorsalis* | AY874302 | HM998979 | HM998933 | AY523722 | HM998962 | DQ347405 | HM998946 | AY523699 | AY948894 |
| *Pipa carvalhoi* | HM998974 | HM998980 | HM998935 | HQ260711 | HM998963 | DQ283922 | DQ284277 |  |  |
| *Silurana tropicalis* | AY874306 | EF535957 | EF433430 | AY523721 | BC088054 | NM_001097334 | CR855729 | AY523697 | AY948891 |
| *Xenopus* | *X. laevis* | *X. laevis* | *X. laevis* | *X. laevis* | *X. laevis* | *X. laevis* | *X. laevis* | *Xenopus* sp. | *X. wittei* |
|  | L19324 | L19325 | HM998930 | X90839 | X05941 | S62229 | J00984 | AY523691 | EF107370 |
| *Hymenochirus boettgeri* | AY583340 | HM998981 | HM998932 | AY523702 | HM998964 | AY323735 | HM998947 | AY523685 | EF107344 |
| *Pseudhymenochirus merlini* | HM998975 | HM998982 | HM998934 | HM998953 | HM998965 | HM998985 | HM998948 |  |  |
| *Pelobates* | *P. cultripes* | *P. fuscus fuscus* | *P. fuscus fuscus* | *P. cultripes* | *P. fuscus fuscus* | *P. cultripes* | *P. fuscus* | *P. cultripes* | *P. cultripes* |
|  | AY323758 | HM998983 | HM998931 | AY523707 | HM998966 | AY323736 | DQ284159 | AY364171 | AY948857 |
| *Duttaphrynus* | *D. melanostictus* | *B. regularis* | *D. melanostictus* | *D. melanostictus* | *D. melanostictus* | *D. melanostictus* | *D. melanostictus* | *D. melanostictus* | *D. melanostictus* |
|  | EU712821 | AY323784 | HM998937 | AY948805 | DQ158317 | DQ283967 | DQ284324 | AY364167 | AY948851 |
| *Hyla* | *H. chinensis* | *H. chinensis* | *H. chinensis* | *H. chinensis* | *H. japonica* | *H. japonica* | *H. chinensis* | *H. meridionalis* | *H. meridionalis* |
|  | HM998976 | HQ260710 | HM998936 | HM998954 | DQ055794 | AY844615 | HM998949 | AY523687 | AY948860 |
| *Microhyla* | *M. pulchra* | *M. pulchra* | *M. pulchra* | *M. ornata* | *Microhyla* sp. | *M. ornata* | *Microhyla* sp | *M. ornata* | *M. ornata* |
|  | EF396093 | EF396134 | EF396021 | AY948806 | HM998967 | AY364383 | DQ284400 | AY364168 | AY948852 |
| *Kaloula pulchra* | AY323772 | AY323790 | EF396015 | EF018030 | HM998968 | DQ284011 | DQ284379 | EF017974 | AY948853 |
| *Fejervarya* | *Fejervarya* sp. | *Fejervarya* sp. | *F. limnocharis* | *F. limnocharis* | *F. limnocharis* | *F. limnocharis* | *F. limnocharis* |  |  |
|  | AY571649 | DQ019526 | HM998938 | HM998955 | HM998969 | DQ458271 | DQ284356 |  |  |
| *Mantella* | *M. madagascariensis* | *M. madagascariensis* | *M. madagascariensis* | *M. madagascariensis* | *M. madagascariensis* | *M. madagascariensis* | *M. aurantiaca* |  |  |
|  | DQ019500 | DQ019532 | HM998940 | HM998957 | HM998971 | AY263284 | DQ284061 |  |  |
| *Polypedates* | *P. cruciger* | *P. maculatus* | *P. cruciger* | *P. cruciger* | *P. cruciger* | *P. megacephalus* | *P. leucomystax* |  |  |
|  | HQ260712 | AY323802 | HM998939 | HM998956 | HM998970 | EU924545 | DQ284079 |  |  |
| *Rhacophorus* | *R. dennysi* | *R. dennysi* | *R. dennysi* | *R. dennysi* | *R. dennysi* | *R. dennysi* | *R. dennysi* | *R. malabaricus* | *R. malabaricus* |
|  | DQ019512 | DQ019547 | HM998941 | HM998958 | HM998972 | EU215575 | HM998950 | AY948769 | AY948848 |
